# Unraveling Chylomicron Retention Disease Enhances Insight into SAR1B GTPase Functions and Mechanisms of Actions, While Shedding Light of Intracellular Chylomicron Trafficking

**DOI:** 10.3390/biomedicines12071548

**Published:** 2024-07-12

**Authors:** Emile Levy, Catherine Fallet-Bianco, Nickolas Auclair, Natalie Patey, Valérie Marcil, Alain Théophile Sané, Schohraya Spahis

**Affiliations:** 1 Azrieli Research Center, CHU Ste-Justine and Department of Nutrition, Université de Montréal, Montreal, QC H3T 1C5, Canada; 2 Azrieli Research Center, CHU Ste-Justine and Pathology & Cell Biology, Université de Montréal, Montreal, QC H3T 1C5, Canada; 3 Azrieli Research Center, CHU Ste-Justine and Pharmacology, Université de Montréal, Montreal, QC H3T 1C5, Canada; 4 Azrieli Research Center, CHU Ste-Justine, Montreal, QC H3T 1C5, Canada; 5 Azrieli Research Center, CHU Ste-Justine and Biochemistry & Molecular Medicine, Université de Montréal, Montreal, QC H3T 1C5, Canada; schohraya.spahis@gmail.com

**Keywords:** Anderson disease, SAR1B GTPase, fat malabsorption, embryo lethality, genetically modified cellular/animal models, gender differences

## Abstract

Over the past three decades, significant efforts have been focused on unraveling congenital intestinal disorders that disrupt the absorption of dietary lipids and fat-soluble vitamins. The primary goal has been to gain deeper insights into intra-enterocyte sites, molecular steps, and crucial proteins/regulatory pathways involved, while simultaneously identifying novel therapeutic targets and diagnostic tools. This research not only delves into specific and rare malabsorptive conditions, such as chylomicron retention disease (CRD), but also contributes to our understanding of normal physiology through the utilization of cutting-edge cellular and animal models alongside advanced research methodologies. This review elucidates how modern techniques have facilitated the decoding of CRD gene defects, the identification of dysfunctional cellular processes, disease regulatory mechanisms, and the essential role of coat protein complex II-coated vesicles and cargo receptors in chylomicron trafficking and endoplasmic reticulum (ER) exit sites. Moreover, experimental approaches have shed light on the multifaceted functions of SAR1B GTPase, wherein loss-of-function mutations not only predispose individuals to CRD but also exacerbate oxidative stress, inflammation, and ER stress, potentially contributing to clinical complications associated with CRD. In addition to dissecting the primary disease pathology, genetically modified animal models have emerged as invaluable assets in exploring various ancillary aspects, including responses to environmental challenges such as dietary alterations, gender-specific disparities in disease onset and progression, and embryonic lethality or developmental abnormalities. In summary, this comprehensive review provides an in-depth and contemporary analysis of CRD, offering a meticulous examination of the CRD current landscape by synthesizing the latest research findings and advancements in the field.

## 1. Introduction

**Figure 1:**
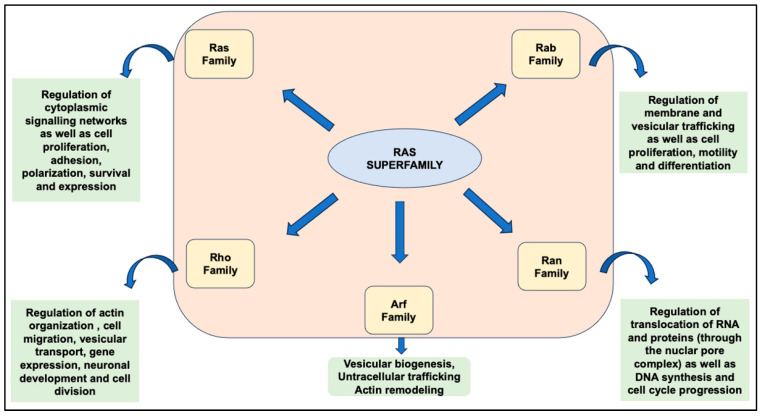
Members of the RAS superfamily and their functions. The RAS superfamily is a group of small GTPase proteins that play crucial roles in various cellular processes, including cell growth, differentiation, and intracellular signaling. The present figure illustrates the key members of the RAS superfamily and examples of their functions, including cell proliferation, differentiation, motility, migration, adhesion, and survival, as well as nucleocytoplasmic transport, actin cytoskeleton organization, and intracellular vesicle trafficking and budding, transport, and fusion with target membranes. Notably, the Arf family are the founding members of Arf-like, Arf-related, and Sar proteins, which have diverse functions in membrane trafficking, cytoskeletal organization, and cell signaling pathways. They regulate processes such as vesicle budding and actin dynamics.

Members of the superfamily known as small GTPases, alternatively called G-proteins or the Ras superfamily, play an integral role in numerous biological processes within cell biology. These proteins (~150) undergo a transformation from an active GTP-bound state to an inactive GDP-bound state following the hydrolysis of GTP to GDP. Categorized based on their structural resemblance, sequence, and intracellular functions, the GTPases within the RAS superfamily are classified into five primary groups: RAS, RHO, RAB, ARF, and RAN [1,2,3,4]. All these small GTP-binding proteins (~20 kDa) convert extracellular signals to various cellular functions (Figure 1).

**Figure 2:**
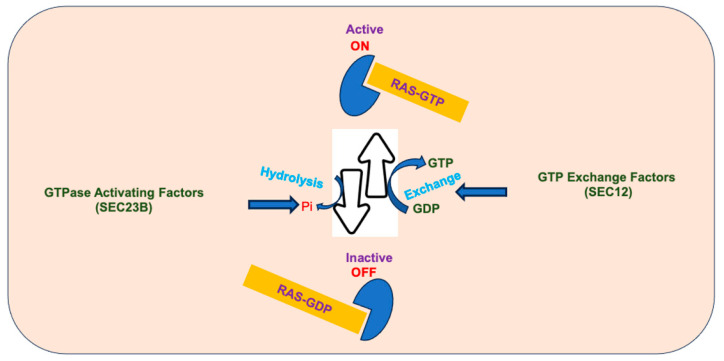
The GTP/GDP cycle ensures control of cellular signaling pathways. RAS is activated upon binding to GTP to promote cell proliferation. The hydrolysis of GTP to GDP and Pi turns off the active form of RAS. An equilibrium is maintained between RAS-GTP and RAS-GDP forms and GTPase activating protein and GTP exchange factor coordinate the relative proportions of each form.

Receptors for various signaling molecules transmit messages to RAS GTPases, which act like binary molecular switches in cell communication. These switches have an “ON” state (active) when bound to GTP, allowing them to interact with downstream proteins for signaling. In their “OFF” state with GDP (inactive), they cannot engage with downstream proteins. GTPases control essential cell functions, like gene expression, cell structure, protein and vesicle transport, and cytoskeletal remodeling in response to the regulation of a wide array of signaling cascades [5,6,7]. Importantly, the molecular switches possess inherent GTPase activity, which is accelerated by GTPase-activating proteins, while guanine nucleotide exchange factors initiate their active state [8] (Figure 2). This review delves into several pivotal questions: How does the guanosine triphosphatase SAR1B regulate the assembly and scission of coat protein complex II (COPII) vesicles? What role does it play in transporting proteins synthesized by the endoplasmic reticulum (ER)? Who are its partners, and through what mechanisms do much larger chylomicrons (CM) and very low density lipoproteins (VLDL) leverage the capacity of COPII for transport? What clinical and metabolic repercussions are observed in the presence of *SAR1B* genetic defects? Does *SAR1B* exhibit distinct roles in the liver and intestine? What experimental models are being developed to enhance our understanding of *SAR1B* defects? Have these endeavors uncovered new functions for *SAR1B*?

## 2. Coat Protein Complex II Functions

**Figure 3:**
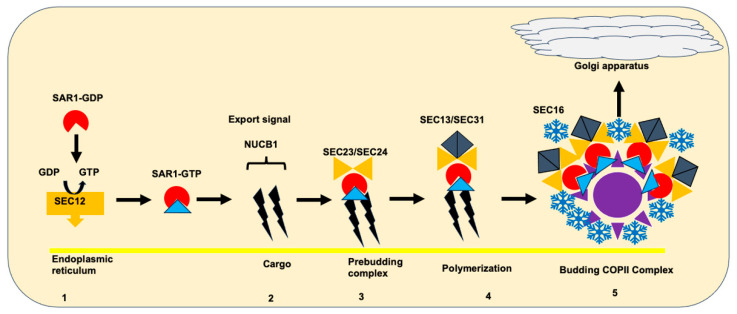
The formation of COPII Transport Vesicle. The building of the COPII complex is a highly regulated process that ensures efficient and selective transport of cargo proteins from the ER to the Golgi apparatus, contributing to the maintenance of cellular homeostasis and proper protein trafficking within the secretory pathway. The assemblage of the COPII vesicle complex occurs in five steps: (1) activation of SAR1 via phosphorylation of SAR1-GDP by SEC12; (2) selection of cargo proteins via the Pro28 N-terminal ER peptide signal of NUCB1 (nucleobindin 1); (3) recruitment of the SEC23/SEC24 subunit to form the inner vesicle layer; (4) recruitment of the SEC13/SEC31 subunit to form the outer vesicle layer; and (5) stabilization of the COPII complex by SEC16 and budding of the vesicle to the Golgi. Importantly, the SEC23 protein (with 5 distinct domains) activates SAR1-GTP hydrolysis to stimulate vesicle transportation.

COPII vesicles play a pivotal role in mediating the exclusive export of newly synthesized cargoes from the ER to the ER-Golgi intermediate complex. Initiated by the activation of SAR1-GTP on ER exit sites through the transmembrane guanine nucleotide exchange factor SEC12, this process involves the insertion of SAR1B into the ER membrane via the pro28N-terminal ER peptide signal of the nucleobindin-1. Subsequent interactions with SEC23/SEC24 form the inner layer, followed by the recruitment of the SEC13/SEC31 complex, constituting the outer layer of COPII vesicles. SEC16 acts as a peripheral protein, serving as a scaffold to stabilize COPII components on the ER membrane, enhances ER exit sites and COPII vesicle formation, and associates with multiple components of COPII vesicles [9,10,11] (Figure 3). Recent studies have unveiled a COPII-regulated tubular network for ER-Golgi protein transport, underscoring the gate-keeping role of COPII at the boundary between the ER and ER exit sites in selecting and concentrating cargo molecules during export.

It can be understood from the points described above that COPII is a crucial cellular process involved in protein sorting and transportation. It is clear, therefore, that when COPII malfunctions or becomes dysregulated, misfolded or unfolded proteins accumulate within the ER, leading to ER stress. The latter triggers a cellular response known as the unfolded protein response, which aims to restore ER homeostasis by reducing protein synthesis, enhancing protein folding capacity, and promoting protein degradation. However, if ER stress persists due to chronic COPII dysregulation, it can overwhelm the unfolded protein response machinery, leading to cell dysfunction and ultimately cell death. In these conditions, impaired protein trafficking and the accumulation of misfolded proteins disrupt the normal functioning of cells and tissues, contributing to the development of human pathological conditions, as is the case in skeletal dysplasia, hematologic abnormalities, and neurological disorders [12,13,14,15,16]. Additionally, COPII deregulation can disrupt the secretion of hormones, growth factors, and other signaling molecules, affecting intercellular communication and tissue homeostasis. This disruption in signaling pathways can contribute to the pathogenesis of metabolic disorders, autoimmune diseases, and cancer [17,18]. In summary, the deregulation of the COPII process can have far-reaching consequences for cellular function and organismal health, contributing to the development and progression of various diseases. Understanding the mechanisms underlying COPII dysfunction is crucial for developing targeted therapeutic strategies to mitigate its adverse effects and restore cellular homeostasis. As SAR1B is a crucial component of the COPII complex, its mutations can evidently affect cargo selection and packaging. Therefore, an appreciation of the importance of SAR1B for COPII function may provide insights into the molecular mechanisms underlying intracellular protein transport and related disorders.

## 3. Protein Partners Regulate the Exit of Transport Vesicles, Especially Those with Cargo Exceeding the Carrier Size

### 3.1. TANGO1

A comprehensive genome-wide screening in Drosophila S2 cells identified Transport And Golgi Organization 1 (TANGO1), also known as Melanoma Inhibitory Activity 3, as a protein involved in ER to Golgi trafficking. TANGO1, a metazoan-specific protein, contains unique structural features, including a non-canonical SH3 domain [19], transmembrane regions, two coiled-coil domains, and a proline-rich domain (PRD). It localizes at ER exit sites with the SH3 domain facing the luminal side and the PRD to the cytoplasmic side. The PRD of TANGO1 interacts with SEC23/SEC24 [20,21], akin to the SEC31’s role in COPII-coated vesicle formation. TANGO1’s SH3 domain interacts with collagen VII, and its knockdown impairs collagen VII export from the ER without affecting general protein transport. Notably, mutations in humans alter the secretion of collagen, leading to various interconnected developmental outcomes [22,23]. Notably, TANGO1’s role as a cargo receptor seems specific to certain sizable molecules [24], as it is not required for collagen I secretion. In vesicle budding assays, TANGO1 does not exit the ER with collagen unlike conventional cargo receptors that exit within COPII-coated vesicles, suggesting a unique mechanism for exporting large cargoes.

### 3.2. cTAGE5

cTAGE5, a tumor-specific antigen (also known as MGEA6) similar to TANGO1, interacts with TANGO1 and binds SEC23/SEC24 at ER exit sites [20,25]. Silencing *cTAGE5* leads to collagen VII accumulation, indicating its role as a TANGO1 co-receptor. Conserved in vertebrates, cTAGE5 has nine pseudo genes in humans [26]. Tissue-specific alternative splicing produces melanoma inhibitory activity 2, expressed only in hepatocytes. The role of melanoma inhibitory activity 2, as an oversized cargo receptor, is unclear, and its cleaved secreted form is implicated in carcinogenesis [27,28,29]. The proposed model suggests that the cTAGE5/TANGO1 complex inhibits SEC13/SEC31 recruitment, facilitating COPII carrier formation for large molecules such as collagen VII.

### 3.3. SLY-SYNTAXIN 18

It has recently been discovered that SLY1 (a member of the SEC1/MUNC18 protein family) regulates SNARE complex assembly through binding to specific SNARE proteins and participating in their proper alignment and interaction. It seems essential for membrane fusion reactions and interacts with TANGO1’s cytoplasmic domain in the presence of a cross linker. SLY1 binds to ER-specific t-SNARE’s SYNTAXIN17 and SYNTAXIN18 [30,31]. In fact, SLY1 facilitates the merging of membranes that surround structures crucial for protein trafficking, including the ER, the Golgi apparatus, and vesicles. This fusion enables the transfer of vesicle cargoes between different cellular compartments. Knocking down *SLY1* or *SYNTAXIN18* specifically hinders pro collagen VII secretion, not affecting collagen I or other ER-exported cargoes [32]. A proposed model suggests that the SLY1-SYNTAXIN18-mediated fusion of recycling membranes, like the ER-Golgi intermediate compartment, enlarges the COPII-mediated carrier, triggered by cTAGE5/TANGO1 action [32,33]. By stabilizing the interactions of SNARE proteins, FLY1 ensures efficient vesicle docking and cargo transport within the cell.

### 3.4. CUL3-KLHL12

A connection has been unveiled between the ubiquitylation of COPII components and the secretion of a large cargo. The depletion of ubiquitin ligase CUL3 (a core component of the ubiquitin E3 ligase that is involved in protein ubiquitination) in mouse embryonic stem cells leads to densely packed cell clusters, indicative of abnormal extracellular matrix deposition [34]. KLHL12, identified as a CUL3 adaptor, exhibits a similar phenotype to CUL3 when knocked down in embryonic stem cells. Notably, CUL3-KLHL12 monoubiquitylates SEC31, promoting the formation of expanded COPII-coated structures (200–500 nm in diameter), where KLHL12 is also present. Importantly, these enlarged COPII structures are crucial for the transport of collagen I and IV [35].

### 3.5. SEDLIN

SEDLIN, also recognized as TRAPPC2, plays a role in the Transport Protein Particle complex, facilitating vesicle tethering during ER to Golgi and intra-Golgi transport [36]. Mutations in the *SEDLIN* gene have been linked to spondyloepiphyseal dysplasia tarda, an X-linked skeletal disorder marked by short stature, a brief trunk, and joint degeneration [37]. The chondrocytes from affected individuals exhibit impaired extracellular matrix molecule secretion. Venditti et al. demonstrated that *SEDLIN* localizes to ER exit sites through interactions with TANGO1 and directly engages with the GTP-bound form of SAR1 [38,39]. *SEDLIN* knockdown results in the accumulation of activated SAR1 at ER exit sites, specifically obstructing collagen I and II secretion from chondrocytes and fibroblasts. The authors propose that SEDLIN regulates the SAR1 cycle to control collagen exit from the ER.

### 3.6. SURF4

The yeast counterpart of surfeit locus protein 4 (SURF4), Erv29p, was discovered through a proteomic analysis of COPII vesicles, and was found to facilitate the secretion and sorting of proteins [40]. In a recent study, SURF4 was highlighted as a key mediator facilitating proprotein convertase subtilisin/kexin type 9 secretion in HEK293T cells, using an innovative experimental strategy merging proximity-dependent biotinylating with CRISPR-mediated functional genomic screening [41]. This finding lends support to a recent report demonstrating the involvement of the *C. elegans* homolog (SFT-4) in facilitating the secretion of yolk lipoproteins and in mediating apolipoprotein (Apo) B secretion in HepG2 cells [42]. In fact, the loss of SURF4 in HepG2 cells resulted in the accumulation of Apo B in the ER, causing impaired Apo B secretion, but the overall secretion process was not universally hindered by the absence of SURF4, suggesting its specific role in the secretion of Apo B [43]. This finding is not surprising since human SURF4 was found to localize to and cycle in the early secretory pathway similar to ER-Golgi intermediate compartment-53, and the silencing of SURF4 together with ER-Golgi intermediate compartment-53 disrupted the Golgi apparatus and led to instability [44]. It is possible that SURF4 functions in coordination with the TANGO1/cTAGE5 complex during cargo enrichment and loading into triglyceride (TG)-rich lipoprotein particles (Table 1).

Overall, these important proteins (i.e., TANGO, cTAGE5, SLY-SYNTAXIN 18, CUL3-KLHL12, SEDLIN, and SURF4) are closely involved in different aspects of the COPII-mediated transport pathway, contributing to the efficient formation, cargo selection, and trafficking of COPII vesicles from the ER to the Golgi apparatus. Collectively, they serve as key mediators in the export process of large cargo molecules (e.g., procollagens, mucins, and even lipoproteins), which surpass the typical size constraints of conventional transport vesicles. They form a network of molecular interactions that regulate various steps of COPII vesicle biogenesis and trafficking to accommodate oversized cargo molecules: (i) TANGO, SEDLIN, and SURF4 are involved in coordinating the assembly of COPII coat components and regulating the budding of COPII vesicles from the ER membrane. TANGO acts as a scaffold protein, facilitating the assembly of COPII components, while SEDLIN and SURF4 interacts with COPII coat proteins and modulate the efficiency of vesicle formation; (ii) cTAGE5 functions as a cargo receptor, interacting with SEC23/24 and facilitating the packaging of specific cargoes into COPII vesicles. Additionally, SEDLIN and SURF4 are involved in cargo selection processes, assisting in the concentration of cargo molecules into nascent COPII vesicles; (iii) SLY-SYNTAXIN 18 participates in vesicle fusion events by interacting with SNARE proteins on COPII vesicles and target membranes, facilitating the fusion of vesicles with their target compartments during membrane trafficking; and (iv) CUL3-KLHL12 regulates COPII vesicle formation by modulating the ubiquitination and degradation of SEC31, a component of the COPII coat complex. This regulatory mechanism helps fine-tune COPII vesicle biogenesis and ER-to-Golgi trafficking.

## 4. CRD: Valuable Lessons from Genetically Engineered Cells and Animals to Determine Whether SAR1B Is a Critical Driver of Disease-Related Genes

Experimental models have been crucial for understanding SAR1B functions and mechanisms. They have allowed researchers to study the CRD caused by the genetic defect, identify the responsible gene, and define gene functions within a complete biological context [45,46,47]. By specifically disrupting a gene, researchers can observe phenotypic effects and determine the precise role of that gene.

(i) Organotypic culture: This technique is known to maintain the complex architecture and cellular diversity of the original intestinal tissue, in addition to modeling human intestinal physiology and pathology. Therefore, the application of CRD intestinal biopsies in organotypic culture emerged as a valuable strategy, unraveling the molecular and cellular intricacies of the disease [48]. Subsequent confirmation of the disease’s underlying etiology involved validating impaired dietary lipid transport and compromised CM delivery.

(ii) Caco-2/15 cell line: This remarkable model was also used since it naturally differentiates into a monolayer of cells resembling mature enterocytes, and is ideal for investigating gut absorption mechanisms. For a better understanding of CRD, intestinal Caco-2/15 enterocytes with abrogation of *SAR1A* and *SAR1B* by the zinc-finger nuclease and/or CRISPR-Cas9 systems were established to delve deeper into the cause–effect relationship between SAR1B expression, CM output, high-density lipoprotein (HDL) production, and lipid metabolism [49,50].

(iii) Zebrafish: As a powerful and versatile tool in scientific research, zebrafish offer insights into genetics, development, reproduction, and disease mechanisms. They were further used to model CRD pathophysiology. *SAR1B* loss-of-function via an antisense oligonucleotide knockdown resulted in poor fat absorption and developmental defects, including abnormal differentiation and the maturation of craniofacial cartilage [51].

(iv) Embryonic mouse model: *SAR1B* loss-of-function was performed in the embryonic mouse cortex through in utero electroporation to examine cortical development [52]. The findings suggest that SAR1B is required for the normal positioning of the cortical neurons during embryonic development.

(v) Genetic engineering of mice: Genetic modifications in cellular and animal models have proven instrumental in exploring *SAR1B* gene functions, shedding light on its roles in various biological processes. These models serve as invaluable tools, advancing our comprehension of molecular and cellular processes in CRD, offering critical insights that may be elusive through alternative methods. For proof-of-principle experiments related to the specific functions of SAR1B, CRD mouse models were developed using the CRISPR-Cas9 editing system by separately introducing a deletion and a point mutation identified in patients [53,54]. The findings clearly enhanced our understanding of SAR1B functions [53,54].

In summary, harnessing genetically modified in vitro and in vivo models provides a potent avenue for gaining fresh insights into diverse biological phenomena occurring in CRD pathogenesis.

### 4.1. SAR1B Functions

#### 4.1.1. SAR1B GTPase Is a Central Factor for COPII Budding, Assembly and Uncoating

SAR1B GTPase is a vital protein highly involved in the movement of newly synthesized proteins within cells. In fact, SAR1B is essential for the formation of transport vesicles that carry newly synthesized proteins from the ER to the Golgi apparatus. Acting as a molecular switch, SAR1B alternates between an active state (GTP-bound) that promotes vesicle formation and an inactive state (GDP-bound). Overall, its facilitating protein transport and its participating in the regulation of the secretory pathway ensure final protein destinations in the cell and proper cellular organization. In a practical way, SAR1B, present in an inactive state in the cytosol, binds to the ER membrane upon activation by SEC12p, its specific guanine nucleotide exchange factor [55,56]. Once activated, SAR1B GTPase then systematically brings together the SEC23/SEC24 and SEC13/SEC31 complexes, facilitating the assembly of the inner and outer layers of the COPII coat, respectively [57]. In their turn, the SEC23/SEC24 and SEC13/SEC31 both act as GTPase-activating proteins [58], inducing GTP hydrolysis on SAR1B, thereby leading to the detachment of SAR1B and to the uncoating process [57]. Of the utmost importance to note that *SAR1B* mutations that impede the exchange of GDP to GTP or GTP hydrolysis, provoke deficiencies in secretion, which underscore the crucial role of GTP turnover [59]. These investigations unequivocally demonstrate the indispensability of SAR1B in COPII vesicle formation [60,61].

#### 4.1.2. Requisite of SAR1B for ER Membrane Deformation and Cargo Selection

To facilitate COPII vesicle generation, the restructuring of the membrane at ER Exit Sites (ERES) is indispensable, with the binding of SAR1B being a pivotal factor in enhancing membrane curvature. SAR1-mediated membrane deformation involves various mechanisms, broadly classified into three approaches: insertion of amphipathic helices, membrane crowding, and scaffolding.

Upon GTP-loading by SEC12, SAR1 undergoes a conformational change, revealing an amphipathic helix at its N-terminus. This helix inserts into the membrane, forming a stable interaction [62]. The deletion of this helix eliminates membrane interaction, impacting vesicle budding efficiency [62,63]. The presence of multiple helices in the outer leaflet induces a global area difference, leading to positive curvature.

Membrane crowding contributes to membrane bending, where numerous SAR1 molecules at the same membrane area collide, and their release coincides with membrane curvature [64]. At high concentrations, SAR1 may form regular arrays, suggesting a role in inducing curvature through scaffolding [65]. Scaffolding refers to a supportive framework aiding in SAR1-mediated membrane deformation mechanisms. This framework might play a supportive role in vesicle formation and maintain the structural integrity of the cellular membrane during specific membrane binding. It is possible that these very well-studied models are operational at different steps of membrane remodeling, but further efforts are still needed to clarify the framework for COPII formation and cargo secretion. In this same context, work is required to provide knowledge on the scission. It is thought that the interplay between SEC12-mediated GTP exchange and COPII-induced GTP hydrolysis is proposed to sustain a high concentration of dynamically cycling SAR1 at the base of the bud (Figure 2). This elevated SAR1 density contributes to the destabilization of the bilayer, ultimately facilitating the membrane necks of coated buds to undergo fission [57].

#### 4.1.3. SAR1B as an Undeniable Key Player in the Uptake and Transport of Exogenous Lipids via CMs

The role of SAR1B in human pathologies has been understood from CRD. Young patients with this syndrome were diagnosed with a malabsorption syndrome characterized by typical features. In addition to steatorrhea, in response to post-consumption of a fatty meal, there was no observable increase in plasma TG, and CMs could not be identified [66]. An analysis of lipoprotein composition revealed normal Apos, elevated phospholipids, and reduced cholesterol (CHOL). Immunoperoxidase localization of Apo B in fasting biopsy specimens exhibited heightened staining of lipid-laden intestinal epithelial cells compared with normal samples. Electron microscopy following a fat load revealed enterocytes containing numerous fat particles, leading to vesiculation of the ER. These particles, resembling CMs morphologically, were found as aggregates of well-individualized lipid droplets within dilated vesicles in the Golgi zone compared with healthy subjects. Notably, they were absent in the interstitium space between adjacent enterocytes and lacteals. An exploration of the plasma lipid status indicated normal fasting TG, hypocholesterolemia, and a deficiency in essential fatty acids and liposoluble vitamins, particularly A and E. Additionally, there was a notable decrease in plasma levels of low-density lipoprotein (LDL), Apos (B, A-I). We concluded that CRD is characterized by fat malabsorption, hypocholesterolemia, and marked intestinal steatosis despite the presence of both plasma and intestinal Apo B. In a subsequent study, jejunal explants of CRD patients were investigated for their ability to synthesize lipids and Apos using labeled substrates such as [^14^C]-palmitate and [^3^H-leucine [48]. TGs and cholesteryl esters were retained in the tissue enterocytes and could not be secreted into the culture medium. Despite the presence of Apo B-48 evidenced by specific antibodies, a defective release of CMs was demonstrated [48]. Confirmation was obtained by substantiating that the defect of CM secretion is not due to Apo B editing or biosynthesis [67]. Therefore, the development of CRD does not stem from the absence of Apo B-48 or its flawed synthesis, as evidenced in our findings on abetalipoproteinemia [66] and hypobetalipoproteinemia [68]. The underlying issue seems to lie in the hindered secretion of CMs, pointing towards a likely deficiency in the final assembly of these molecules and/or their delivery.

Later, it was determined in collaborative studies that SAR1B, a single polypeptide of 198 amino acids, is impaired in CRD through the sequencing of *SARA2*, the gene coding for *SAR1B* [69,70]. All 10 patients within the six families exhibited mutations on both alleles of *SARA2*, which abolish the production of functional SAR1B [70]. Missense mutations in *SAR1B* are the predominant cause of CRD, with a significant number of these mutations being located within the GDP or GTP binding site of SAR1B [69,70,71,72]. Also illustrated previously, computational analysis and sequence alignment are helpful to explain the functional impairment of mutated proteins [71]. Importantly, the *SARA2* gene is more prominently expressed in various other tissues (e.g., skeletal muscle, liver, heart, kidneys, and placenta) other than the intestine [70], which may be indicative that clinical implications related to these tissues may be present in CRD [73]. Several clinical data do not align with a presumed correlation between genotype and phenotype [56,74].

#### 4.1.4. Importance of SAR1B in Maintaining HDL Status

Patients with CRD experienced a significant impact on the HDL fraction, as its CHOL content accounted for only 22% of the values observed in the control group [48]. The marked decrease in HDL-CHOL is particularly noteworthy and could indicate a diminished production of HDL by the small intestine in individuals with CRD. Given that the intestine plays a pivotal role in the synthesis and secretion of HDL, as well as Apo A-I (the primary Apo of HDL), contributing approximately 50% of total plasma Apo A-I, we sought to test the hypothesis that *SAR1B* deficiency adversely affects intestinal HDL output. Our experiments, utilizing micellar [^3^H]-CHOL and [^35^S]-methionine and intestinal Caco-2/15 cells characterized by *SAR1B* knockout using the zinc finger nuclease technique, verified the impaired ability of enterocytes to secrete HDL and Apo A-I, respectively [50]. Another hypothesis considered was that SAR1B downregulates the protein expression of ATP-binding cassette transporter A1 (ABCA1), a facilitator of CHOL efflux, to Apo A-I acceptor, thus influencing HDL formation. This situation is reminiscent of patients with Tangier disease, a disorder linked to *ABCA1* mutations, resulting in defective CHOL transfer to an extracellular Apo A-I acceptor, an essential protein for HDL maturation. Our prediction held true, as evidenced by low CHOL efflux in response to *SAR1B* silencing, attributable to diminished ABCA1 expression compared with control cells [50].

These data in intestinal Caco-2/15 cells are in line with findings obtained using a stable isotope kinetic study in two patients with CRD [75]. In response to an infusion of ^13^C-leucine for 14 h, the averaged production rate of HDL-Apo A-I was lower (68%) while the fractional catabolic rate (FCR in day^−1^ or pool/day) was higher in the patients (1,5-fold) in comparison with healthy individuals [75]. Therefore, the diminished rate production and raised rate catabolism of Apo A-I may explain the low plasma Apo A-I of CRD patients.

#### 4.1.5. SAR1B as a Key Protein for Metabolic Homeostasis

The emphasis on the modulation of intestinal lipid transport by the SAR1B protein has been demonstrated in transgenic mice via SAR1B overexpression [76]. The close correlation between SAR1B overexpression and increased fat absorption underscores the pivotal role of this protein in directing/moving ApoB-48-containing CMs from the ER to the Golgi apparatus, and their release from enterocytes. It can be inferred that, under circumstances where CM-TG flux is heightened due to an increased dietary fat load, the augmentation of SAR1B becomes essential. This enhancement is necessary to facilitate the accommodation of CM cargos, working in conjunction with crucial proteins that regulate intracellular assembly in TG-rich lipoproteins.

Another noteworthy discovery in transgenic mice was the exhibition of metabolic abnormalities upon exposure to a western diet [76]. The presence of abundant SAR1B expression was found to exacerbate body and adipose weight, hepatic steatosis, elevated circulating lipids, and insulin insensitivity. Prolonged exposure to heightened intestinal fat absorption due to SAR1B abundance could potentially result in greater TG accumulation in insulin-responsive tissues over time, exacerbating insulin insensitivity. In addition, the induction of *SAR1B* in transgenic mice subjected to a high-fat diet led to elevated plasma fatty acids. This increase could hinder glucose uptake in various tissues, potentially triggering insulin resistance. Such phenomena may particularly compromise hepatic lipid homeostasis, ultimately leading to liver steatosis. Of note, the use of these transgenic mice underscores the significance of SAR1B in organs engaged in lipid transport including the liver, the intestine, the skeletal muscle, and the heart [76,77].

**Figure 4:**
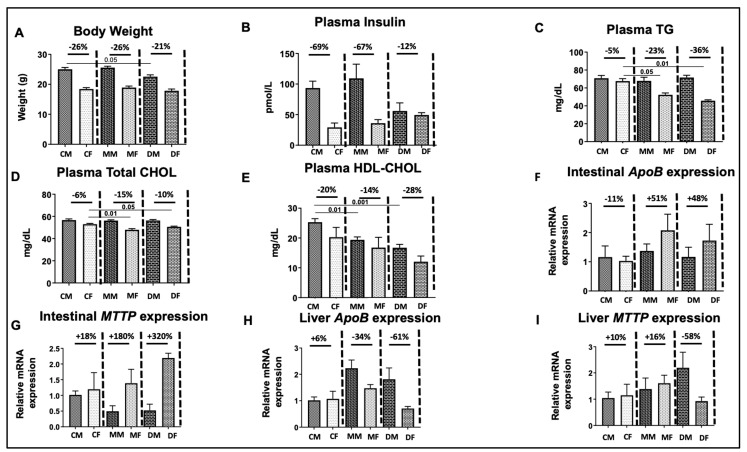
*SAR1B* genetic defects and gender-related differences. After stratifying the animal groups by sex, the genetically modified animals exhibited more significant gender divergences in (**A**) body weight, (**B**) plasma insulin levels, (**C**–**E**) plasma lipid profile, as well as (**F**,**G**) intestinal and (**H**,**I**) liver *Apo B* and *MTTP* gene expressions. Results represent the means ± SEM of 10–13 mice in each group. CM = control male, CF = control females, MM = *Sar1b^mut/+^* males, MF = *Sar1b^mut/+^* females, DM = *Sar1b^del/+^* males, and DF = *Sar1b^del/+^* females. This figure is a new supplementary analysis obtained as part of previous data [53,54].

We have also succeeded in generating a mouse model with either a targeted deletion or mutation in *SAR1B* through the CRISPR-Cas9 system [78]. To assess the metabolic impact of these genetic modifications, the mice were subjected to an 8-week diet consisting of 60% fat. Notably, the control mice exposed to a high-fat diet exhibited a significant increase in body weight, augmented adipose tissue, enlarged liver size, and the development of insulin resistance as evidenced by elevated plasma insulin levels and HOMA-IR index [53]. On the other hand, animals with the *SAR1B* mutation or deletion showed less pronounced effects of the high-fat diet (Figure 4). This comprehensive approach allows us to explore the nuanced metabolic responses associated with *SAR1B* alterations, shedding light on its role in various physiological aspects.

#### 4.1.6. SAR1B as an Important Protein for Cholesterol Metabolism

The distribution of CHOL across eukaryotic cells and particularly in plasma membrane plays a pivotal role in cellular equilibrium [79]. The importance of CHOL in this intracellular site is highlighted by the fact that (i) the plasma membrane contains 60–80% of the cell’s free CHOL and approximately 35–45% of its lipid content [80]; and (ii) alterations in CHOL concentrations within a membrane may dramatically alter the physical properties of a membrane, affecting diverse processes such as signal transduction, membrane trafficking, or the function of integral proteins such as ion channels [81].

The secretory pathway plays a crucial role in intracellular protein/lipoprotein transport, commencing from the ERES and progressing to the Golgi apparatus before eventual extracellular cargo release. CHOL is a vital component for the efficiency of ER-to-Golgi trafficking [82]. Recent investigations have highlighted a substantial enrichment of CHOL at ERESs [83]. This notable presence of CHOL at ERES is likely attributed to its facilitation in sorting specific cargo proteins at these sites, given its ability to segregate lipids and proteins into subdomains within a continuous bilayer [84]. Importantly, Weigel et al. concluded that the rates of cargo entry into and exit from ERES could be explained by differential affinity of cargo for COPII or cargo receptors and/or the attraction of the cargo to the ERES apparent CHOL-rich lipid environment [83]. To better understand the relationship between SAR1B-directed COPII vesicles and CHOL metabolism, heterozygous *SAR1B*-deficient mice (*Sar1b^mut/+^* and *Sar1b^del/+^* mice) were challenged with high-fat diet and compared with normal mice (on Chow diet) for 8 weeks [53]. From the outset, fluctuations in plasma CHOL concentrations and lipoproteins led us to hypothesize that CHOL metabolism in the intestine and liver, the two organs working together to regulate CHOL levels in the body, was altered in heterozygous *SAR1B*-deficient mice (Figure 4). Measurement of their CHOL content showed a marginal increase in liver CHOL content in female *Sar1b^mut/+^* mice subjected to a high-fat diet, contrasting with a reduction in male *Sar1b^del/+^* mice compared with their respective controls. Gene expression analysis showed alterations in crucial genes involved in CHOL endocytosis (proprotein convertase subtilisin/kexin type 9, LDL-receptor], transport [Niemann–Pick C1-like 1, scavenger receptor class B type I, ABCG8, Microsomal triglyceride transfer protein (MTTP)], synthesis (3-hydroxy-3-methylglutaryl coenzyme A reductase, sterol regulatory element-binding protein-2), and reverse CHOL transport (ABCA1, Liver X receptor α) [53,54]. Noteworthy distinctions were observed between controls and heterozygous mice, as well as between the liver and intestine, *Sar1b*^del/+^ and *Sar1b*^mut/+^ genotypes, and males and females under the two dietary conditions (Figure 4). Overall, these findings highlight the interplay between *SAR1B* aberrations and fat feeding-mediated metabolism.

#### 4.1.7. SAR1B as a Guardian Shielding against Oxidative Stress

There is a growing interest in understanding how lipids influence oxidative stress (OxS). The question of whether fat accumulation in various organs acts as a contributory factor to OxS development has captured the attention of numerous researchers. Under normal physiological conditions, the radical scavenging system, comprising antioxidant enzymes like superoxide dismutase, glutathione peroxidase (GPx), and catalase, effectively eliminates excess reactive oxygen species to maintain cellular redox balance. However, when reactive oxygen species production surpasses the capacity of cellular antioxidant defenses, oxidative damage ensues.

In this context, our hypothesis posited that *SAR1B* mutations impede the intracellular transport of exogenous lipids, leading to their accumulation in the secretory pathway and triggering significant OxS. This hypothesis proved accurate, as evidenced by the notable increased level of malondialdehyde, a biomarker of lipid peroxidation and OxS, in *SAR1B* deleted Caco-2/15 cells, indicating substantial lipid peroxidation [49]. Further investigation into the expression levels of GPx, a critical protein in defending against OxS, revealed a significant reduction in GPx protein levels in *SAR1B* deleted cells compared with controls [49]. Our attention then turned to NF-E2-related factor-2 (NRF2), a nuclear transcription factor pivotal in the redox homeostatic gene network. *SAR1B*-deleted cells exhibited a considerable decrease in NRF2 protein expression [49]. This concurrent decline in NRF2 supports the idea of compromised antioxidant defense.

Our findings also disclose a spontaneous and considerable surge in lipid peroxidation, accompanied by reduced GPx levels, indicating a decline in the breakdown of harmful compounds. The heightened intracellular lipids due to *SAR1B* deletion may overwhelm the antioxidant defense system, contributing to increased OxS in intestinal cells, consistent with prior studies linking lipid accumulation and reactive oxygen species generation [85]. Additionally, heightened OxS may be attributed to the increased mitochondrial combustion of fatty acids, as excessive fatty acid oxidation is known to induce OxS and reduce antioxidant defenses [86]. It is widely accepted that mitochondria are the primary source of intracellular ROS, as the electron transport process consumes approximately 85% of the cell’s oxygen [87].

In summary, *SAR1B* silencing results in OxS likely stemming from intracellular lipid deposition. In CRD patients, OxS may be exacerbated by the deficiency of liposoluble antioxidant vitamins, a direct consequence of fat malabsorption. Ultimately, lipotoxicity and OxS may compound the complications observed in CRD patients, underscoring the urgency for effective therapeutic strategies. Furthermore, further investigations are needed to elucidate the direct impact of SAR1B on OxS generation.

#### 4.1.8. SAR1B as a Safeguard against Inflammation

Substantial evidence strongly supports the association between lipid accumulation and the initiation and progression of inflammation across various organs. This correlation is frequently observed in the context of metabolic disorders such as non-alcoholic fatty liver disease or atherosclerosis [88,89,90]. The mechanisms behind this connection are multifaceted: (i) Elevated lipid levels can induce lipotoxicity and OxS, triggering inflammatory responses [91,92]; (ii) Lipid accumulation can exert stress on cellular machinery, leading to cellular injury or death [93]; (iii) Dying cells release signals that activate immune responses, thereby contributing to inflammation; (iv) Cells laden with lipids can release pro-inflammatory molecules, including cytokines and chemokines, attracting immune cells to the sites of lipid accumulation and promoting inflammation [94,95]; and (v) Lipids can directly interact with immune cells, activating them and further stimulating inflammation [96].

Considering the intracellular lipid accumulation resulting from *SAR1B* disruption, a comprehensive evaluation of inflammation became imperative. Notably, the expression levels of tumor Necrosis Factor-α were significantly elevated in *SAR1B*-disrupted cells at both the gene and protein levels [49]. To explore the potential activation of nuclear factor-kappaB (NF-κB), a pivotal regulator of proinflammatory cytokines, we examined NF-κB p65 protein expression in Caco-2/15 cells. Genetically modified cells exhibited a robust increase in NF-κB p65 expression. The substantial elevation of the NF-κB/I-κB ratio affirmed the activation of NF-κB in conditions of *SAR1B* deletion [49].

#### 4.1.9. SAR1B as a Defender against ER Stress

To investigate the potential association between *SAR1B* mutations and a strain on ER protein quality control mechanisms, we assessed ER stress in both the gut and liver. As is generally recognized, the ER constitutes a distinctive and constantly changing intracellular network where proteins undergo synthesis, folding, maturation, and transportation [97]. The unfolded protein response operates through its three branches to monitor the conditions in the ER lumen and communicate this information across the lipid bilayer to the cytoplasm, signaling cellular stress. Overall, the components work together to regulate cellular responses to ER stress, helping to restore protein-folding homeostasis and promoting cell survival. However, the dysregulation of the unfolded protein response due to prolonged ER stress is implicated in various diseases [98,99].

Our findings indicate that *SAR1B* defects led to alterations in the gene expression of ER stress biomarkers, particularly in the intestines of high-fat diet-fed mice [53]. Specifically, mRNA levels of key ER-stress factors exhibited significant increases compared with their respective controls. This suggests a potential activation of the unfolded protein response, aimed at restoring protein homeostasis through the activation of protein kinase RNA-like ER kinase, inositol-requiring enzyme-1, and transcription factor 6 sensors. The latter are crucial for stimulating downstream pathways (such as G protein-coupled receptor 78) necessary to attenuate protein synthesis while enhancing ER-associated folding and degradation [100,101]. Therefore, it is reasonable to propose that *SAR1B* gene defects may induce ER stress and trigger the unfolded protein response, indicating that SAR1B plays a role beyond its crucial function in TG-rich lipoprotein secretion.

#### 4.1.10. SAR1B Is Essential for Embryonic Development

Recently, extensive efforts were undertaken to generate mice carrying either a targeted deletion or mutation analogous to that found in human SAR1B in CRD patients using the CRISPR-Cas9 system. Despite the phenotypic health and fertility observed in heterozygous *Sar1b^del/+^* and *Sar1b^mut/+^* mice, successive intercrossing over several generations failed to yield homozygous *Sar1b^del/del^* and *Sar1b^mut/mut^* mice, as evidenced by the genotyping of postnatal offspring. These knockout experiments revealed that genetic *SAR1B* alterations in mouse lead to embryonic lethality in homozygotes, emphasizing the crucial role of SAR1B in mouse embryonic development [53].

To investigate the possibility that stillborn homozygous pups were subject to maternal predation due to their genetic background, pregnant animals’ cages were daily inspected, and any deceased pups were removed and genotyped. All deceased pups were found to be homozygous for the deletion, indicating late-gestation lethality and the inability to produce viable homozygous mice. However, no living neonatal or deceased pups with mutations on both alleles were observed, suggesting early embryonic lethality [53].

Confirmation of the absence of live births for *Sar1b^del/del^* and *Sar1b^mut/mut^* homozygotes was corroborated by Mendelian frequency ratios in subsequent studies. These disparities may be attributed to embryonic lethality, similar to animal models with abetalipoproteinemia [102]. Even the gene disruption of the APOBEC-1 complementation factor, responsible for C-to-U editing of the nuclear Apo B mRNA, led to embryonic lethality [103].

**Figure 5:**
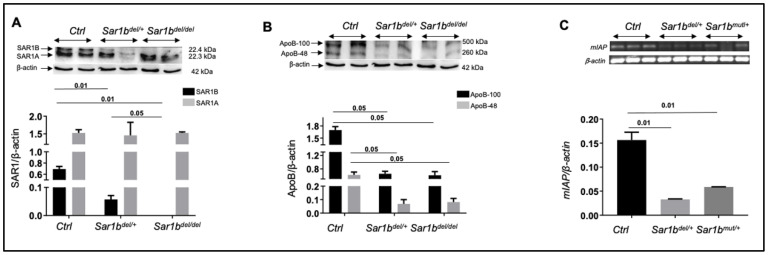
Embryonic expression of SAR1 and Apo B proteins and alkaline phosphatase gene. E18.5 embryos of a *Sar1b^del/+^* pregnant mouse from intercross of two *Sar1b^del/+^* mice were collected and genotyped. Afterwards, two whole embryos from each genetic background were homogenized in cold PBS buffer containing antiproteases. Then, total protein extracts were subjected to 4–20% SDS-PAGE gradient gel and electroblotted onto a same nitrocellulose membrane. The membrane was subsequently reacted with anti-SAR1 (**A**) (provided by Dr Randy Schekman, University of California, Berkeley), anti-Apo B (**B**), and anti-β-actin as loading control using the BLUeye Prestained Potein Ladder, Tris-Glycine 4-20% as a quality control for the molecular weight. In parallel, total RNA (1 μg) from three flash-frozen jejunums of different genetic backgrounds was used for cDNA synthesis in 5X All-In-One RT Master Mix. PCR was then performed with primers for mouse intestinal alkaline phosphatase (m*IAP*) (forward: TCCAGCTGAAGAGGAGAAC; reverse: TTAGGATCCTGGTGGCTGTC) and mouse actin gene (forward: GACAGGATGCAGAAGGAGATTACTG; reverse: CCACCGATCCACACAGTACTT) with Taq DNA polymerase. PCR products were run against 1.5% agarose gel and ethidium bromide reactive bands were visualized with ChemiDoc imaging system (**C**). Bands densitometry was calculated with Image Lab 6.0 software (Bio-Rad, Montreal, CA, USA). Mice are usually from the same litter and are segregated after genotyping. Results represent the means ± SEM of two to three specimens as for preliminary investigation. The original gels This figure is a new analysis obtained as part of previous data [53,54].

To pinpoint the period of embryonic death during development, DNA was collected from embryos at 9.5, 13.5, and 18.5 days of gestation. Homozygous embryos (*Sar1b^del/del^* and *Sar1b^mut/mut^* mice) were identified at all these gestational periods. Under these conditions, it is conceivable that homozygotes with the *SAR1B* deletion succumb just before delivery, while those with the *SAR1B* mutation died but reached full term. Therefore, the total loss of *SAR1B* function affects embryonic development and explains the reason why no one of genotyped pups from *SAR1B* heterozygote mating pairs displayed a homozygote deletion or mutation of the *SAR1B* allele. The transfer of lipids from the mother to the fetus is a crucial aspect in embryonic development. They are deposited in the yolk sac and need active mechanisms to be delivered via lipoproteins to the embryo. Defects in *SAR1B* as illustrated in Figure 5A probably affect the embryonic absorption of different lipid classes, including essential fatty acids and liposoluble vitamins, causing early embryonic lethality. This assumption receives indirect backing first from our findings exhibiting Apo B-48 lessening in embryos (Figure 5B), and second from previous studies, which reported that genetic aberrations of *MTTP* and *Apo B*, expressed by the yolk sac and implicated in lipid release, result in premature embryonic lethality [102]. Since intestinal alkaline phosphatase (IAP), a small intestinal brush border enzyme, is essential for optimal lipid digestion and absorption, as well as overall intestinal function and health [104], its protein expression was also examined in homozygous embryos. Obviously, *SAR1B* deletion and mutation culminate in a marked decrease in IAP (Figure 5C). Although further studies are required to define the mechanisms for this association in homozygous embryos, the fall of IAP may deteriorate embryo development given its multifaceted roles such as the emulsification of fats, the activation of digestive enzymes, the facilitation of micelle formation, intestinal barrier permeability, the detoxification of lipopolysaccharides, immune function, and the modulation of inflammation and intestinal microbiota [104,105,106].

**Figure 6:**
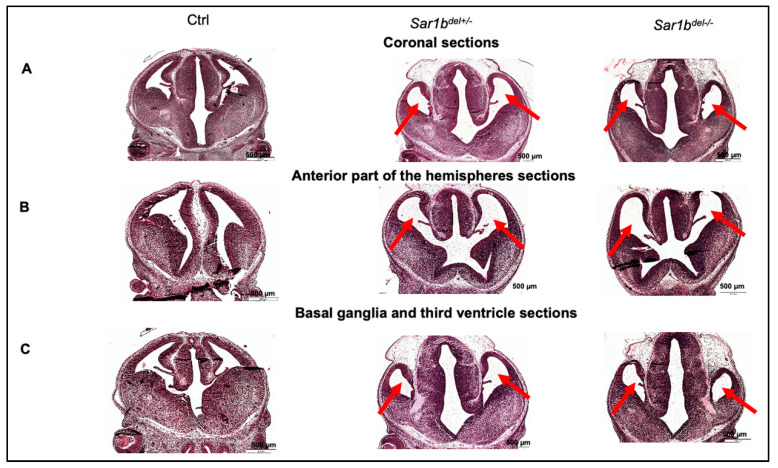
Coronal sections of embryonic mice brain showing dilated ventricles in anterior part of the hemispheres, in basal ganglia and in the third ventricle of SAR1B animal models. In coronal sections of embryonic mice (13.5 days), the presence of dilated ventricles in animal models, particularly in *del/+* and *del/del* mice (as indicated by red arrows) is observed. As ventricles are fluid-filled cavities playing important roles in cerebrospinal fluid circulation and brain development, the observation of dilated ventricles in *Sar1b^del/+^* and *Sar1b^del/del^* mice implies disruptions or alterations in normal brain development processes. Particularly, dilation is predominant in the anterior part of the lateral ventricles (**A**), in ganglia (**B**), and in the third ventricle (**C**), and is identical in heterozygotes and homozygotes. These abnormalities may lead to impaired neurogenesis, altered neuronal migration, or defective formation of brain structures, resulting in the observed dilation of the ventricles. Overall, the presence of dilated ventricles in embryonic mice brain sections may serve as a morphological indicator of potential brain developmental abnormalities in these animal models. This figure is a new supplementary analysis obtained as part of previous data [53].

Not only did the homozygous embryos (*Sar1b^del/del^* and *Sar1b^mut/mut^*) display no signs of macroscopic abnormalities at E18.5 in our studies, but the brain morphology also appeared normal [53,54]. However, in coronal sections of embryonic mice at 13.5 days, the lateral and third ventricles appeared enlarged in *Sar1b^del +/−^* and *Sar1b^del−/−^* mice, compared with controls (Figure 6). In both cases, this increased ventricular volume was associated with a reduced thickness of the hemispheric mantle and a more restricted development of the basal ganglia compared with controls. These findings suggest alterations in the normal processes of brain development. The inadequate development of the mantle and basal ganglia may result from delayed or insufficient neurogenesis and impaired migration, leading to delayed development and maturation mostly affecting forebrain structures, and is responsible for ventricular enlargement.

Importantly, embryos of mice with homozygous *APOB* deficiency exhibit significant neurodevelopmental abnormalities, including exencephalus and hydrocephalus [107]. Likewise, embryos with homozygous *MTTP* knockout experience mortality during mid gestation [108]. Moreover, the zebrafish model of *SAR1B* deficiency was characterized with irregular differentiation and maturation of craniofacial cartilage (in association with deficiencies in procollagen II secretion), and the absence of specific neuroD-positive neurons in the midbrain and hindbrain [51]. Finally, through experiments involving in utero electroporation, the downregulation of *SAR1B* in the developing cerebral cortex hinders the radial migration and axon elongation of cortical neurons [52]. Clearly, further studies are needed to unveil the role and function of SAR1B during embryonic development. For the time being, our hypothesis concerning the lethality of homozygous embryos is that *SAR1B* mutations/deletions impair yolk sac lipoprotein secretion, and culminate in deficient nutrition of the developing embryo, as is the case for MTTP and Apo B irregularities. For example, the deficiency of vitamin E causes fetal resorption and embryonic exencephalus in pregnant rats [109,110]. Additional investigations are also necessary to dissect the precise role of SAR1B in brain development in rodents and higher animals.

### 4.2. SAR1B Mutations Affect Differently the Small Intestine and Liver

While all the studies have consistently indicated the incapacity of the intestine to secrete CM in CRD, there is noteworthy documentation of plasma VLDL resulting from hepatic release. This intriguing observation suggests a differential response to congenital *SAR1B* mutations between the intestine and the liver. One plausible consideration is the potential influence of the size disparity between CM and VLDL particles. Alternatively, the divergence in the responses of the intestine and liver could stem from the distinct presence of Apo B-48 in CM and Apo B-100 in VLDL [49]. These apos serve as molecular recognition signals, possibly engaging in binding interactions with other factors that, in turn, direct the vesicles along distinct cellular itineraries. The intricate interplay of these factors raises questions about the molecular mechanisms underlying the divergence in vesicle secretion pathways and warrants further investigation to elucidate the nuanced dynamics at play.

### 4.3. SAR1B Genetic Defects and Gender-Related Differences

An integral aspect was explored in genetically modified mice expressing the *SAR1B* deletion and mutation [54]: the presence of phenotypic differences between males and females. Notably, CRD in mice exhibited distinct manifestations based on gender, with pronounced differences across various parameters compared with wild-type mice (Figure 4). Firstly, gender disparities were more evident, showcasing variations in body weight, plasma insulin, TG, CHOL, and HDL-C, as well as hepatic expressions of *APO B* and *MTTP* (Figure 4). Males exhibited higher values for these parameters, whereas the opposite trend was observed for intestinal *APOB* and *MTTP*. Furthermore, male and female *Sar1b*^mut/+^ and *Sar1b^del/+^* mice displayed divergences in both the pattern and magnitude of these variables (Figure 4).

These gender differences should not be overlooked, especially in the current era of personalized medicine. This is particularly significant since numerous studies have highlighted gender differences in lipid and lipoprotein metabolism [111]. Notably, these distinctions were more pronounced in response to both chow and high-fat diets, as elucidated in a subsequent study [53]. For instance, notable differences emerged in anthropometric measurements, insulin resistance parameters, and lipid accumulation in the jejunum and liver.

Despite the growing interest in understanding the influence of sex and gender on health, congenital malabsorption diseases have received limited attention in this regard. Nonetheless, these conditions may exert considerable influence on prevalence, onset, treatment response, and prognosis. Additional efforts are imperative to discern gender-specific differences, unraveling the intricacies of SAR1B. Addressing this knowledge gap is not only vital for advancing our understanding of fundamental biological processes but also essential for developing targeted interventions and treatments that consider individual and gender-specific variations.

### 4.4. Cooperation of SAR1B and SEC23B to Activate CM and VLDL Secretion

As previously noted, the SAR1 protein in its GTP-bound form attaches to the ER membrane and summons the SEC23/SEC24 heterodimer, forming the prebudding complex. Subsequently, this complex attracts the outer coat consisting of SEC13/SEC31 heterotetramers, thereby finalizing the COPII coat structure. There is an integral collaboration between SEC23B and SAR1B. SAR1B initiates the assembly of the COPII coat and SEC23B acts as a GTPase-activating protein to activate SAR1B. In addition, SEC23B is instrumental in selecting and packaging cargo proteins into the vesicles. Evidently, the two proteins cooperate to ensure accurate sorting while facilitating their delivery to various cellular compartments [112].

The interdependence between SAR1B and SEC23 is crucial for the formation of COPII-coated vesicles, facilitating the transportation of proteins, including CM, from the ER to the Golgi apparatus. The intricate nature of the SAR1B–SEC23 interaction underscores its significance, as any disruption or malfunction in the SEC23 protein can profoundly impact SAR1B, leading to disturbances in the processing and transport of lipid molecules. Our hypothesis posits that abnormalities in SEC23 functionality may compromise its efficient interaction with SAR1B, hindering its role in cargo recognition and packaging. In other words, disruption, especially within the intra-enterocyte trafficking of CM-containing vesicles, may significantly affect the proper secretion of TG-rich lipoproteins in intestinal enterocytes. Furthermore, as functional COPII components are crucial for the release of VLDL in hepatocytes, their defects may hamper VLDL output.

To test this assumption, we conducted a preliminary experiments involving the silencing of endogenous SEC23B expression in Caco-2/15 and HepG2 cell lines. For comparative analysis, we also invalidated *SAR1B* in both cell types. Utilizing Lipofectamine 2000 (Invitrogen), cells were transfected with 50 pmol/well of scrambled or specific siRNA-*SEC23B* or siRNA-*SAR1B*, following the manufacturer’s protocol. After 72 h of transfection, HepG2 and Caco-2/15 cells were incubated for an additional 24 h with [^14^C]-oleic acid and [^35^S]-methionine to measure the major lipid classes and Apos, respectively, released into the media.

Our findings show that silencing *SEC23B* resulted in a significant decrease in the secretion of TG, Apo B-100/48, and VLDL from HepG2 cells, as well as TG, Apo B-48, and CM from Caco-2/15 cells. These findings provide empirical support for our hypothesis, underscoring the pivotal role of the SAR1B-SEC23 interaction in lipid transport processes. Indeed, mitigating potential disruptions in the SAR1B-SEC23 interaction is crucial for maintaining the integrity of intracellular transport processes, especially in the context of CM (intestine) and VLDL (liver) trafficking. Any dysregulation in this intricate molecular machinery may have implications for lipid metabolism and related physiological processes. Further research in this area is essential for elucidating the detailed mechanisms and potential therapeutic interventions to address such disruptions.

## 5. Conclusions and Future Directions

The identification of the defective *SAR1B* gene associated with a CRD has proven to be a transformative milestone. This breakthrough not only provides insight into the genetic basis of the disorder but also serves as a foundation for unraveling the underlying mechanisms and developing valuable tools for diagnosis and ongoing patient monitoring.

Once the *SAR1B* gene responsible for CRD has been pinpointed, investigators could delve into its normal function within the body and the aberrations that occur when it is mutated or dysfunctional. Knowing the specific *SAR1B* gene allows engineering mouse models with precision through introducing mutations or alterations into *SAR1B* genes in mice to replicate the genetic basis of the human disorder. This precision is essential for accurately establishing the proof of concept and disease-relevant phenotypes (development of symptoms and pathological features like those seen in human patients), thereby recapitulating the genetic context of the disorder in mouse models. Both the cellular and mouse models provided a platform to gain insights into the underlying disease mechanisms by focusing on dysfunctional cellular processes and molecular pathways.

Moreover, the use of *SAR1B* mice has been instrumental in advancing our understanding of both gender-specific aspects. The animal models allowed the investigation of specific biological processes and physiological differences between male and female mice in a controlled environment. They have also contributed to studying the intricacies of lethal embryonic development. So far, the examination of morphological, histological, and molecular changes in homozygous *SAR1B* embryos did not disclose organ malformations, disruptions in crucial signaling pathways, and abnormal developmental processes.

In summary, CRD, abetalipoproteinemia, and hypobetalipoproteinemia are all rare genetic disorders that affect lipid metabolism and result in abnormalities in lipoprotein metabolism. Despite sharing some similarities, each disorder has distinct pathophysiological mechanisms and clinical presentations. CRD is characterized by fat malabsorption, the accumulation of lipids in the enterocytes of the small intestine, and the deficiency of essential fatty acids and fat-soluble vitamins (e.g., A, D, E, and K) with an impact on other organ systems. Clinical manifestations of CRD typically present in infancy or early childhood and include chronic diarrhea, steatorrhea, failure to thrive, and developmental delays, as well as neurological, hepatic, and cardiological abnormalities. CRD is caused by mutations in the *SAR1B* gene, resulting in defective CM transport release CM from the ER to the Golgi apparatus.

Cell and animal models of rare diseases play a crucial role in advancing our understanding not only of the diseases themselves but also of the broader implications of their defective genes in cellular physiology. Such is the case with the CRD experimental models, which provide valuable platforms for advancing our understanding of both the physiological role of SAR1B and the underlying mechanisms of diseases associated with its dysfunction. In fact, mutations/deletions in the *SAR1B* gene have significantly advanced our understanding of both the physiological role of SAR1B and the underlying mechanisms of diseases associated with its dysfunction. The scientific community has gained valuable insights into the specific role of SAR1B in intracellular CM trafficking and secretion processes, oxidative stress, inflammation, ER stress, embryogenesis, neurological development, lipoprotein metabolism, and liver abnormalities. Further efforts will help identify novel therapeutic targets, and ultimately improve the management and treatment of *SAR1B*-related diseases.

## Figures and Tables

**Table 1:** Partners of SAR1B for lipoprotein transport.

Partners	Gene (kb)	Protein (kDa)	Amino Acids (nb)	Subcellular Location	Gene Location	Functions
**TANGO1**	69.91	214	1907	Golgi	1q41	Required for protein secretion; Binds to COPII subunits for cargo loading
**cTAGE5**	1.5	110	395	Plasma membrane	14q21.1	Export of large pre-chylomicrons and pre-VLDLs
**SLY-SYNTAXIN 18**	21	42	335	ER, Golgi	4p16.3	Targeting and fusion of Golgi-derived retrograde transport vesicle with ER
**CUL3-KLHL12**	135.25	89	768	Nucleus, Golgi	2q36.2	Regulates COPPII size for ER-Golgi transport; Controls ubiquitination of GEF
**SEDLIN**	68.12	16	140	ER	Xp22.2	Vesicular transport from ER to Golgi
**SURF4**	34.72	30	269	ER	3q34.2	Recruits cargos into COPII; Cargo receptor for APOB/APO A-1 lipoproteins; Regulates lipid transport; Promotes PCSK9 secretion; Synergizes with SAR1B
**SAR1**	68.12	22	198	ER, Golgi	5q31	Vesicular transport from ER to Golgi; Selection of cargo and assembly of COPII; Synergizes with the cargo receptor Surf4; Exports lipoproteins from ER; Regulates lipoprotein deliver; Maintains lipid homeostasis

Gene and protein molecular weights, amino acid contents, subcellular and gene locations, as well as their functions are noted. COPII, Coat Protein Complex II; VLDL, Very Low Density Lipoprotein; ER, Endoplasmic; GEF, guanine nucleotide exchange factor; Apo B, Apolipoprotein B; Apo A-1, Apolipoprotein A1; PCSK9, Proprotein Convertase Subtilisin/Kexin type 9.

## Data Availability

Not applicable.
